# Anti-inflammatory effect of lamivudine on ulcerative colitis

**DOI:** 10.1016/j.bbrep.2025.102317

**Published:** 2025-10-22

**Authors:** Xiaoyu Chen, Huijuan Wang, Yingqiang Liu, Wei Zhang, Zhangfa Song

**Affiliations:** Department of Colorectal Surgery, Sir Run Run Shaw Hospital, Zhejiang University School of Medicine, Hangzhou, China

## Abstract

Ulcerative colitis (UC) is a non-specific inflammatory bowel disease characterized by ulcers and erosions in the colonic mucosa, leading to a chronic cycle with alternating periods of remission and exacerbation. Ascribed to its elusive etiology, primary therapy is limited to symptomatic treatment. Consequently, there is a pressing need to identify novel therapeutic agents for UC. Lamivudine, a nucleoside analog known for its antiviral properties and Long Interspersed Nuclear Element 1 (LINE-1) inhibitory effects, was investigated for its potential anti-inflammatory role in UC. Thus, this study aimed to investigate the anti-inflammatory effect of lamivudine on DSS (Dextran Sulphate Sodium)-induced ulcerative colitis (UC) in mice.

Briefly, the mice were randomly assigned to the control group, the model group, or the lamivudine group. Following the administration of the predefined treatments, the mice were monitored, and the disease activity index (DAI) score was calculated. Moreover, histological analysis was performed to examine the efficacy of lamivudine in alleviating colitis-induced damage to the large intestine. Additionally, hematoxylin-eosin (HE) staining was performed to detect pathological alterations in intestinal tissues. Finally, the levels of IL-6, IL-17, IL-10, and TNF-α were measured using enzyme-linked immunosorbent assay (ELISA).

The results revealed that the DAI score and enteritis pathological score were significantly lower in the lamivudine group compared to the model group (P < 0.05). Additionally, lamivudine significantly down-regulated the expression of the inflammatory factors IL-6, IL-17, and TNF-α in intestinal tissues and concomitantly up-regulated the expression of the anti-inflammatory factor IL-10. Lastly, lamivudine significantly attenuated the symptoms of colitis and the level of intestinal inflammation in mice and exerted anti-inflammatory effects.

## Highlights

• Lamivudine reduces disease activity and pathological scores in DSS-induced colitis mice.
• Lamivudine modulates cytokine levels, reducing pro-inflammatory and increasing anti-inflammatory factors.
• LINE-1 expression is elevated in UC patients, suggesting a potential therapeutic target.
• This study provides preliminary evidence supporting lamivudine as a novel treatment for UC.

## 1 Introduction

As is well documented, ulcerative colitis (UC) is a chronic nonspecific inflammatory bowel disease (IBD) hallmarked by recurrent episodes of remission and relapse. It is typically manifested by abdominal pain, diarrhea, mucoid pus, bloody stool, tenesmus, etc. Notably, IBD patients are also at an elevated risk of developing colorectal cancer, with the risk being correlated with the severity and duration of relapses [1,2]. While its underlying etiology has not yet been fully elucidated, it is universally recognized that UC develops as a result of an aberrant and aggressive inflammatory response. At present, primary treatment is limited to symptomatic treatment aimed at inducing or maintaining remission using amino salicylic acid anti-inflammatory drugs, immunosuppressants, or colon resection. Although lifestyle modifications and pharmacological interventions can relieve the symptoms of IBD, a definitive cure is not achievable by the majority of patients [3], highlighting the need for novel therapeutic agents.

Therefore, this study aimed to identify effective drugs and approaches for UC treatment. Long Interspersed Nuclear Element-1 (LINE-1) is the only autonomous retrotransposon in the human genome. Beyond its role in genome evolution, aberrant LINE-1 activity has been increasingly linked to pathological inflammatory conditions. Its activation can lead to DNA double-strand breaks and, crucially, the accumulation of its nucleic acid products (RNA and cDNA) can act as endogenous ligands that trigger innate immune pathways, such as the cGAS-STING axis, thereby perpetuating inflammation. Lamivudine, a nucleoside reverse transcriptase inhibitor (NRTI), is well-established for its antiviral efficacy. Importantly, beyond viral reverse transcriptase, NRTIs like lamivudine have been demonstrated to potently inhibit the reverse transcriptase activity encoded by LINE-1 [4]. This property positions lamivudine as a candidate drug for investigating LINE-1-driven pathologies. To the best of our knowledge, there are currently no reports on the use of Lamivudine for the treatment of ulcerative colitis. The dextran sodium sulfate (DSS)-induced colitis animal model in IBD research offers several advantages over other chemically induced experimental models owing to its rapidity, simplicity, and reproducibility [5]. Therefore, this model was used to investigate the effect of lamivudine on UC and lay an experimental reference for the application of lamivudine in the prevention or treatment of ulcerative colitis.

## 2 Materials and methods

### 2.1 Clinical samples

From August 2021 to February 2022, 13 fresh diseased colon tissue specimens from patients with UC confirmed by pathology and 13 normal colon tissue specimens were obtained to analyse the expression of LINE-1. The study was conducted under the accordance with ethical guideline of Declaration of Helsinki. Ethics Committee of the Sir Run Run Shaw Hospital, Zhejiang University (20210714-100) had approved these studies. All patient tissue samples used in this study were obtained with informed consent from the participants. Each participant was fully informed about the study's purpose, procedures, and their rights.

### 2.2 Animals

Experimental animals: 36 SPF C57BL/6 male mice, aged 5 weeks old and weighing 17–19 g, were purchased from Shanghai Lingchang Biotech Company (Certificate No.2018-0003). Housing conditions strictly adhered to the guidelines outlined in GB14925, with mice having ad libitum access to food and water throughout the experiment. To alleviate suffering, suitable living conditions were ensured, including clean cages and appropriate temperatures, and carbon dioxide anesthesia was used in the sacrifice of mice. The study protocols were approved by the Ethics Committee of Zhejiang University.

### 2.3 Experimental reagents

Lamivudine tablets (H20030518) were sourced from GlaxoSmithKline Pharmaceutical (Suzhou) Co., Ltd. DSS was purchased from TdB, Sweden, tissue cell lysate and PMSF were procured from Shanghai Biyuntian Biotechnology Co., Ltd., isoflurane was obtained from Shenzhen Rivard Life Technology Co., Ltd., and formaldehyde solution was obtained from Shanghai Cologne Chemical Co., Ltd.

### 2.4 RNA extraction and quantitative RT‒PCR

Total RNA was extracted from tissues using RNeasy Reagent (Invitrogen, Carlsbad, CA, USA). Reverse transcription was then performed with reagents provided by TaKaRa (Tokyo, Japan) to convert the total RNA into cDNA. Specific primers were utilized to detect the mRNA levels of the target gene, using this cDNA as the template.

### 2.5 UC model preparation

The mice were acclimatized for one week after purchase, with unrestricted access to food and water. Then, they were randomly assigned to 3 groups, each consisting of 12 mice: control group (CTL group), DSS model group (DSS group), and lamivudine intervention group (LAM group). The CTL group had ad libitum access to pure water, whereas the remaining groups were administered 2.5 % dextran sodium sulfate (DSS) for 7 days to induce UC. From the 8th day, mice in the CTL group and the DSS group received pure water, and those in the LAM group were treated with lamivudine solution (100 mg/35 mL normal saline) at a dose of 100 mg/kg once daily for 7 days by oral gavage.

### 2.6 DAI reporting

Body weight, stool consistency, and hematochezia were documented daily to calculate the disease activity index (DAI) [6] and assess disease severity. DAI has a maximum value of 12 and was determined based on three parameters (weight loss, stool consistency, and occurrence of fecal blood), each with a score ranging between 0 and 4, with higher scores reflecting more severe disease conditions.

The scoring criteria are as follows:

Score 0: There is no weight loss, the stool is normal, and there is no occult blood (negative).

Score 1: The weight loss is between 1 and 5 %, the stool is normal, and there is no occult blood (negative).

Score 2: The weight loss is between 5 and 10 %, the stool is loose, and there is occult blood (positive).

Score 3: The weight loss is between 10 and 20 %, the stool is loose, and there is occult blood (positive).

Score 4: The weight loss is more than 20 %, there is diarrhea, and there is visible bleeding.

### 2.7 Pathological examination

After 7 days of intragastric administration, the mice underwent a 24-h fasting period, during which they had access to only water and were subsequently anesthetized with isoflurane. The diseased colon tissue, about 5 cm from the anus, was sequentially excised, fixed in 4 % paraformaldehyde, dehydrated, and paraffin-embedded. Afterward, 4 μm thick tissue sections were prepared using a rotary microtome and mounted on slides. The sections were stained with hematoxylin and eosin (H&E) and microscopically analyzed, and the colon histology score was determined based on the scoring system [7]. The colon histology score was determined based on the following criteria:

Score 0: There is no inflammation, no inflammatory infiltration range, and no damage.

Score 1: Mild inflammation, with the inflammatory infiltration in the mucosal layer, and 1/3 damage.

Score 2: Medium inflammation, with the inflammatory infiltration in the mucosa and submucosa, and 2/3 damage.

Score 3: Severe inflammation, with the inflammatory infiltration in the muscle layer or through the intestinal wall, and loss of both crypt cells and epithelial cells.

### 2.8 Detection of inflammatory factors by ELISA

Blood samples were collected from the eyeballs of mice and centrifuged at 3000 r/min for 10 min at a temperature of 4 °C. Next, the supernatant was collected and stored at 20 °C for biochemical analysis. The levels of IL-6, IL-17, IL-10, and TNF-α in the colonic tissue of mice in each group were detected following the instructions of the ELISA kits.

### 2.9 Statistical analysis

Graphpad Prism7 software was employed for statistical analyses. Data were expressed as mean ± SD, while group comparisons were performed using One-way ANOVA. P < 0.05 was considered a significant difference, and P < 0.01 was considered a highly significant difference.

## 3 Results

### 3.1 Expression of LINE-1 is elevated in UC patients and DSS-induced colitis mice

**Fig. 1:**
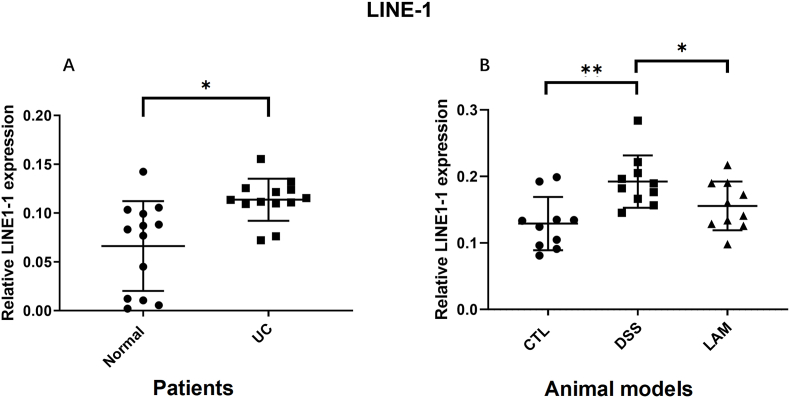
Elevated LINE-1 expression in UC patients and a murine colitis model. Comparison of LINE-1 expression levels in colon tissues between (A) UC patients and healthy controls, and (B) DSS-treated mice and control or Lamivudine-treated mice.

The expression of LINE-1 was analyzed in colon tissues from 13 patients with ulcerative colitis (UC) and 13 healthy individuals. As shown in Fig. 1A, LINE-1 expression was significantly higher in UC patients compared to normal controls (*P* < 0.05). Similarly, in the dextran sulfate sodium (DSS)-induced murine model of colitis, LINE-1 expression was also markedly up-regulated compared to the control group (Fig. 1B). Crucially, treatment with lamivudine (LAM group) significantly down-regulated the DSS-induced overexpression of LINE-1 (Fig. 1B), demonstrating its inhibitory activity on LINE-1 in the context of colitis. These results establish a foundation for the therapeutic application of lamivudine in UC and indicate that its mechanism of action may involve the suppression of LINE-1.

### 3.2 The degree of colon inflammation was lower in the LAM group

**Fig. 2:**
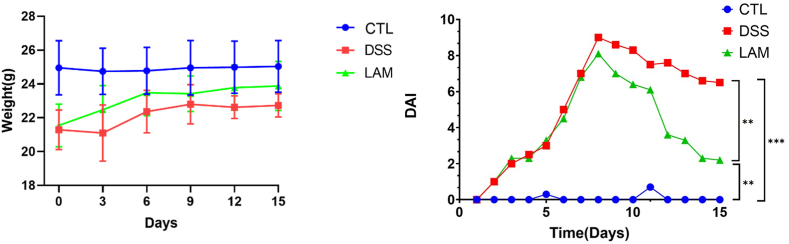
Effect of LAM on Weight loss and DAI (disease activity index) of UC mice.

As displayed in Fig. 2, the DAI score of the CTL group remained consistent throughout the experiment. Conversely, the DAI score was higher in the DSS group from day 5 onwards, indicating the successful establishment of the model. As from the 8th day of intragastric administration, the DAI score of the LAM group progressively decreased.

### 3.3 The degree of tissue destruction was lower in the LAM group

**Fig. 3:**
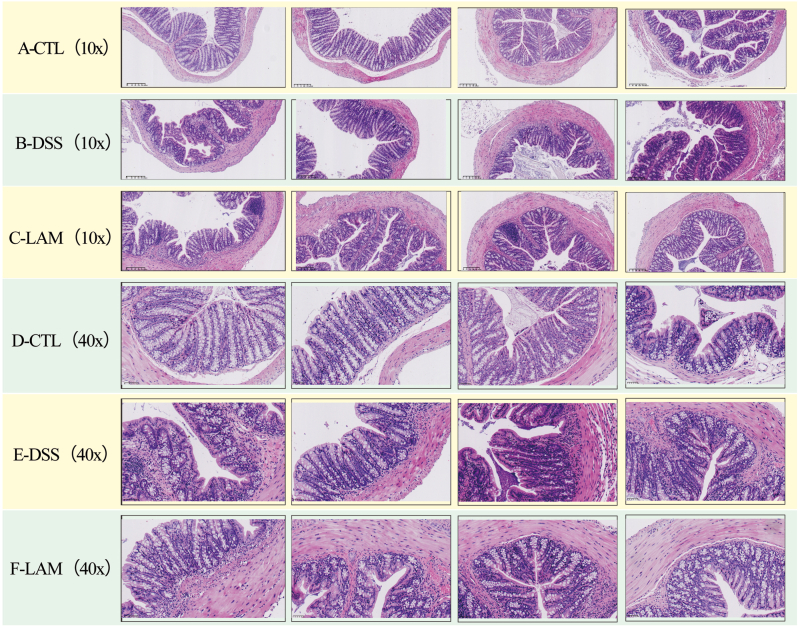
H&E staining of colon tissue shows that, compared to the CTL group with intact tissue and the DSS group with severe inflammatory changes, the LAM group exhibits a better condition with less severe mucosal injury, an intact structure, and minimal inflammatory cell infiltration. 40x samples (line D, E, F) are zoomed version of 10x samples (line A, B, C). Scale bars are located at the bottom left of each image and are presented in the metric system. For 10x images, the value of the scale bar is 200 μm; for 40x images, the value of the scale bar is 50 μm.

H&E staining of the colon sections was performed to assess tissue damage (Fig. 3) where the 40 × samples (row D, E, F) are magnified versions of the 10 × samples (row A, B, C).In the CTL group, the colonic mucosal epithelial tissue of mice appeared intact, with well-defined structures such as the intestinal mucosal layer and the muscular layer and the presence of crypts and goblet cells. Moreover, the glands were uniformly distributed without inflammatory cell infiltration (Fig. 3A–CTL&D-CTL). On the other hand, the colonic mucosal layer of mice in the DSS group was thin, the glands were unevenly distributed, and the extensive infiltration of inflammatory cells extended to the muscular layer, indicative of typical inflammatory changes. Notably, the damaged area of most colon tissues exceeded 50 % of the entire colon. The muscular layer was significantly thickened due to fibrosis and was infiltrated with a large number of inflammatory factors in the mucosal and submucosal layers (Fig. 3B–DSS&E-DSS). Conversely, mucosal injury was less severe in the LAM group. In addition, the colon tissue structure was intact, with visible goblet cells, a uniform crypt arrangement, a marginally thickened muscle layer, and minimal inflammatory cell infiltration (Fig. 3C–LAM&F-LAM).

Comparative analyses were carried out based on histological scores, The analysis exposed that the histological colitis score in the CTL group was 0, which was significantly lower than that of the DSS group (P < 0.01). Likewise, the histopathological score of the lamivudine intervention group was significantly lower than that of the DSS group (P < 0.05, 0.01). Of note, the significantly lower score of the LAM group compared to the DSS group indicated the effectiveness of LAM in suppressing DSS-induced UC injury (see Table 1).

**Table 1:** Histological colitis score (Mean ± SD).

Rating items	CTL group	DSS group	LAM group
**Inflammatory severity**	0	2.6 ± 0.35^##^	1.8 ± 0.23∗∗^#^
**Inflammatory infiltration range**	0	2.7 ± 0.41^##^	2.2 ± 0.19∗^##^
**Crypt damage**	0	3.3 ± 0.87^##^	1.7 ± 0.44∗∗^#^
**Total score**	0	8.6 ± 1.63^##^	5.7 ± 0.86∗∗^##^

Note: Compared with the DSS group, ∗P < 0.05,∗∗P < 0.005, compared with the normal control group ^#^P < 0.05,^##^P < 0.005.

### 3.4 Effect of lamivudine on the expression of inflammatory factors in colon tissue of mice

**Fig. 4:**
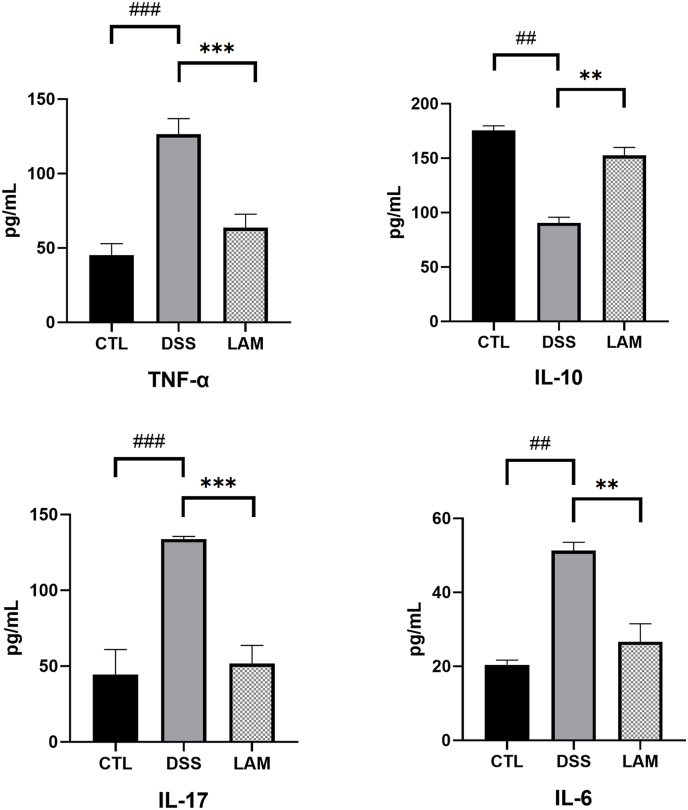
**Expression levels of cytokines by ELISA in the colonic tissues of mice indicated that lamivudine can down-regulate pro-inflammatory factors.** Note: Compared with the DSS group, ∗P < 0.05,∗∗P < 0.01, ∗∗∗P < 0.001compared with the normal control group, ^#^P < 0.05,^##^P < 0.005, ^###^P < 0.001.

The expression of inflammatory factors such as IL-6, IL-10, IL-17, and TNF-α in the colon tissue of mice was detected by ELISA. As portrayed in Fig. 4, the levels of IL-6, IL-17, and TNF-α were significantly higher in the DSS group compared to the CTL group (P < 0.01), whereas the level of IL-10 was significantly lower (P < 0.01). At the same time, the levels of IL-6, IL-17, and TNF-α were significantly lower in the LAM group compared to the DSS group (P < 0.01), whilst the level of IL-10 was significantly higher (P < 0.01). These results collectively implied that the administration of Lamivudine significantly alleviated the symptoms of UC via regulating the imbalance between pro-inflammatory and anti-inflammatory cytokines.

## 4 Discussions

To date, the pathogenesis of ulcerative colitis remains unknown. Earlier studies concluded that it is related to genetic factors. For example, UC patients typically have a family history of the disease, and its incidence is higher in monozygotic twins than in dizygotic twins. Previous studies also identified common antigens and genetic indicators in afflicted individuals. In contrast, other studies postulated that viruses or bacteria, such as cytomegalovirus and Helicobacter, may trigger the onset of UC [8]. The autoimmune theory has been widely accepted, positing that recurrent infections by pathogens invading the intestinal wall and human colonic epithelial cell proteins drive the synthesis of antibodies, complements, cytokines, immune complexes, and immune lymphocytes with killing effects in the intestinal epithelium that promote and aggravate inflammation. Furthermore, alterations in the composition of the intestinal flora, hallmarked by a decrease in the abundance of beneficial bacteria and an increase in that of harmful bacteria, destroy the intestinal mucosa and eventually culminate in ulcerative colitis [9,10].

Lamivudine is a new nucleoside analog that has been extensively administered in the clinical setting. Importantly, it has demonstrated outstanding anti-HBV activity, with minimal toxicity and mild adverse reactions [11]. Lamivudine can be metabolized into lamivudine triphosphate (L-TP) in HBV-infected cells and healthy cells. Following this, L-TP is embedded into viral DNA in the form of cyclic adenosine monophosphate through the action of hepatitis B virus (HBV) polymerase, thereby suppressing viral DNA synthesis. Herein, the expression level of Line-1 was higher in UC patients than in healthy individuals, signaling that Lamivudine, a Line-1 inhibitor, exerts therapeutic effects in UC patients. Therefore, further exploration into its efficacy is warranted.

In order to determine the role of Lamivudine in the prevention or treatment of ulcerative colitis, this study evaluated its effect in DSS-induced UC model mice. The results of the DAI score uncovered that lamivudine relieved symptoms such as weight loss, loose stool, and bloody stool in mice with enteritis, with lamivudine exerting a more significant effect in mice with chronic enteritis compared to those with acute enteritis. Besides, the results of the pathological score highlighted that lamivudine protected the integrity of the intestinal mucosa and epithelium, alleviated damage to crypt cells, and decreased inflammatory cell infiltration and inflammation. The results of ELISA showed that lamivudine reduced the serum levels of the pro-inflammatory factors IL-6, IL-17, and TNF-α and increased the level of the anti-inflammatory factor IL-10. Taken together, these findings highlight the potential of lamivudine for the treatment of UC.

Dysregulation of proinflammatory cytokines is a feature of inflammatory bowel disease (IBD). According to prior investigations, proinflammatory cytokines such as IL-1β, IL-6, IL-17, and TNF-α play a key role in the pathogenesis of IBD. Noteworthily, the dynamic imbalance between anti-inflammatory and proinflammatory cytokines is closely related to the occurrence and progression of inflammatory bowel disease [12]. IL-1β, IL-6, and IL-17 are central pro-inflammatory factors that can promote the activation of T cells and B cells, induce the release of various inflammatory cytokines, and exacerbate intestinal tissue damage in UC patients [13]. TNF-α is one of the most important pro-inflammatory factors in the body and can directly kill or inhibit target cells, activate the NF-κB signaling pathway [14], up-regulate the expression of pro-inflammatory factors, activate the systemic inflammatory cascade, and promote disease progression [15]. The anti-inflammatory properties of IL-10 have been established in studies involving gene-knockout mouse models [16]. The results of this study demonstrated that the serum levels of IL-6, IL-17, and TNF-α were significantly increased in mice in the DSS group, whereas the level of the anti-inflammatory factor IL-10 was significantly decreased. Comparatively, the serum levels of IL-6, IL-17, and TNF-α were decreased to varying degrees in mice in the LAM group, whilst the level of IL-10 was significantly increased. Another key finding of this study is that we not only confirmed elevated LINE-1 expression in UC patient tissues but also, more importantly, observed the same phenomenon in the DSS-induced mouse model of colitis. This suggests that aberrant LINE-1 activation may be a conserved and critical event in the pathogenesis of UC. The underlying mechanism may involve LINE-1 retro transposition activity causing genomic DNA damage, or its RNA/cDNA components acting as endogenous danger signals that activate innate immune pathways, thereby initiating or exacerbating intestinal inflammation Based on the aforementioned results, we speculate that the anti-inflammatory action of Lamivudine is mediated by down-regulating the expression of pro-inflammatory factors and concurrently up-regulating the expression of anti-inflammatory factors, thus reversing the imbalance between anti-inflammatory factors and inflammatory factors.

In summary, the main contributions of this paper are as follows:

1) This is the first study to demonstrate the anti-inflammatory protective effect of lamivudine on ulcerative colitis in mice.
2) A possible mechanism by which lamivudine attenuates intestinal injury and regulates cytokine levels was identified.
3) This study provided an experimental basis for the application of lamivudine in the prevention or treatment of ulcerative colitis.

However, this study has limitations that cannot be overlooked. To begin, the results of this study were based on short-term trials involving a small sample size of animals, and only a single dose of lamivudine was tested, future dose-response studies are warranted to determine the optimal therapeutic dosage. Furthermore, a comprehensive evaluation of safety and effectiveness based on long-term intervention is lacking. Therefore, large-scale clinical controlled trials are necessitated to elucidate the mechanism underlying the effect of lamivudine in UC patients.

## CRediT authorship contribution statement

**Xiaoyu Chen:** Writing – original draft, Visualization, Software, Resources, Project administration, Methodology, Investigation, Formal analysis, Data curation, Conceptualization. **Huijuan Wang:** Writing – review & editing, Visualization, Resources, Formal analysis, Data curation. **Yingqiang Liu:** Writing – review & editing, Validation, Supervision, Methodology, Investigation, Data curation. **Wei Zhang:** Visualization, Software, Resources, Methodology, Formal analysis. **Zhangfa Song:** Writing – review & editing, Validation, Resources, Project administration, Methodology, Funding acquisition, Conceptualization.

## Declaration of competing interest

The authors declare that there are no financial or personal relationships with other people or organizations that could inappropriately influence or bias their work. No funding support, gifts, consulting fees, equity, or other forms of compensation were received from any organization or institution that might have an undue influence on the research results or the publication of this paper.

## Data Availability

Data will be made available on request.
